# Progress in Medicine

**Published:** 1899-06-10

**Authors:** 


### Progress in Medicine.

### DISEASES OF THE RESPIRATORY SYSTEM.

**Bronchitis.**—Hare[1] advises the administration of emetics in urgent cases of bronchitis. Their use is well known and recognised in cases of croup, where it is found that severe symptoms are relieved, the hoarse voice becomes natural, and the fever abates. Similarly, in diphtheria, false membranes—which the patients cough and cough in vain and utterly exhaust themselves to get quit of—are readily brought up by the action of vomiting, to the immense relief of the sufferer. In suffocative bronchitis, too, the effects of emetics are sometimes magical. Twenty-two grains of ipecacuanha in an ounce of water may be given; it is found that in ten or fifteen minutes the patient vomits, and brings up a huge quantity of that tenacious mucus which he was before vainly endeavouring to cough up. The breathing is thereby much relieved, and the patient is able to lie moderately low in bed and to get some sleep. Quite contrary to what those who have had no experience of the plan suppose, the system rallies instead of being more depressed under the action of the remedy.

**Treatment of Coughs with Heroin.**—Dreser made numerous experiments with heroin, which is a derivative of morphine, and found that it had a more profound sedative effect upon respiration than morphine itself. It was ten times more powerful than codein, and at the same time very much less injurious. Floret then tried the substance on patients, and found it to be an exceptionally prompt and useful drug to allay pains in the chest and coughs in both acute and chronic catarrhal inflammations of the respiratory tract. Manges[2] has recently used the drug in the treatment of coughs. It was well borne in the majority of cases, whilst in a few disagreeable after-effects were experienced, but these were milder and less frequent than with morphia or codeia. The aged bear the drug poorly. In allaying coughs the remedy was very prompt and efficacious in a large number of cases. These cases included acute and chronic bronchitis, emphysema, bronchiectasis, phthisis, pleurisy, and pneumonia. In some of the cases the relief was most surprising, being obtained where codein and other drugs had failed. In purely neurotic coughs the results were not so good. The dose is one-twentieth of a grain, which may be increased to one-sixth. The drug is insoluble in water; it may be rubbed up with sugar and given in powder, or dissolved in water by the addition of a few drops of acetic acid.

**Asthma.**—Gee,[3] in the Lumleian lectures, discusses the nature of asthma from two points of view—its anatomical character and its symptoms. In regard to the first, signs of bronchitis and emphysema are always found, whereas no other lesion is constant. Hence we must assent to the proposition that either these constitute its morbid anatomy, or it has no morbid anatomy. The symptoms of an attack of asthma might be due either to a pure affection of the nervous system, or to some gross material change in the lungs, or to a combination of the two. It is conceivable that a contraction of the bronchial musculature might cause dyspnœa, but we must make the reservation that there is no proof of this assumption. Further, such a cause is inadequate to explain the catarrh which manifestly attends most, if not all, asthmatic seizures. It has been supposed that the condition might be due to a spasm of the inspiratory muscles, whereby the chest is in a state of distension; but it is much more likely that this is due to expiratory debility, and that the extreme inflation characteristic of the attack is due to incessant forced inspiratory efforts to make the most of what little room for fresh air can be provided by forced expiration. It is concluded, therefore, that asthma is not due simply to nervous influences.

The argument in favour of the view that asthma is due to gross changes in the lungs is analogical. Asthma may be compared and contrasted with several diseases which resemble it in certain respects, and of which we have more accurate knowledge than of asthma itself. There is, firstly, acute pulmonary catarrh of children, the chief characteristics of which are the rapidity with which the dyspnœa becomes extreme, little or no fever, the short duration—two or three days—of the malady, and the great tendency to recur. Most of the children have suffered from eczema, and most have come from families prone to asthma, bronchitis, and gout. Secondly, spasmodic croup. In this complaint there certainly is an element of laryngitis, for the attack is preceded by hoarseness, and the intervals are not free from the same symptoms. The glottis has never yet been seen during an attack, but it seems improbable that the dyspnœa can be due to glottic spasm lasting several hours, very incomplete, and never passing over into a thorough laryngismus and killing the patient. Again, this spasmodic croup, so called, has a very great resemblance to the peculiar form of bronchitis above-mentioned. It seems, then, probable that it is due to a sudden and temporary swelling of the mucous membrane of the larynx. Thirdly, there is paroxysmal coryza. In this complaint it can be seen that the mucous membrane of the nasal fossæ is swollen, especially the cavernous tissue over the turbinate bones. In many cases paroxysmal coryza is followed by asthma, which suggests the idea of a spreading of the catarrhal affection downwards along the respiratory tract. Hay fever, again, which usually assumes the form of a coryza, sometimes becomes an asthma. There are thus several affections of the respiratory tract which are closely allied to asthma, and which constitute together with asthma a special class of diseases, characterised by sudden onset, short duration, liability to recur, whilst in the interval the patient is more or less free from signs of disease. The tendency to the disorders is hereditary and runs in families; many patients have a tendency to two or more of these complaints. In those members of this group of diseases in which the affected part can be seen there is swelling of the mucous membrane, followed by more or less secretion. The conclusion is that asthma is merely a peculiar form of bronchitis, and that there is no need for any hypothesis of spasm in order to explain the phenomena of the disease.

[1] Hare, Pract., Mar, 1899. [2] Manges, New York Med. Jour., Nov. 26, 1898. [3] Gee, Lancet, Mar. 25, 1899.

